# Protective Behavior and West Nile Virus Risk

**DOI:** 10.3201/eid1109.041184

**Published:** 2005-09

**Authors:** Mark Loeb, Susan J. Elliott, Brian Gibson, Margaret Fearon, Robert Nosal, Michael Drebot, Colin D'Cuhna, Daniel Harrington, Stephanie Smith, Pauline George, John Eyles

**Affiliations:** * McMaster University, Hamilton, Ontario, Canada;; † Ontario Ministry of Health and Long-term Care, Toronto, Ontario, Canada;; ‡ Halton Region Health Department, Oakville, Ontario, Canada;; § Health Canada, Winnipeg, Manitoba, Canada

**Keywords:** West Nile virus, risk factors, serosurvey, seroprevalence, protective behavior, mosquito repellent, dispatch

## Abstract

We conducted a cross-sectional, household survey in Oakville, Ontario, where an outbreak of West Nile virus (WNV) in 2002 led to an unprecedented number of cases of meningitis and encephalitis. Practicing >2 personal protective behavior traits reduced the risk for WNV infection by half.

Little is known about risk factors for infection with West Nile virus (WNV). Data about the effect of personal protective behavior traits recommended by public health agencies, such as wearing long sleeves and long pants, using mosquito repellent, and avoidance of mosquito areas, are sparse (*1*).

A household-based seroprevalence survey in Oakville, Ontario, where a large outbreak of WNV occurred in the summer of 2002, allowed us to assess modifiable risk factors for WNV infection. Oakville is located in Halton, a region that had the highest reported incidence of clinical WNV infection in Ontario in the 2002 season. Sixty cases (58 confirmed and 2 probable) occurred in a population of almost 400,000, with onset during the months of August and September 2002 (Figure 1). A peak in dead crow sightings in Halton (600 per week) occurred 5 weeks before the peak in human cases. Within this region, most cases occurred in south Oakville, in the L6L and L6K forward sortation areas (FSAs, i.e., the first 3 digits of the postal code) (Figure 2). We hypothesized that personal protective and source-reduction behavior would be associated with reduced risk for WNV infection.

**Figure 1:**
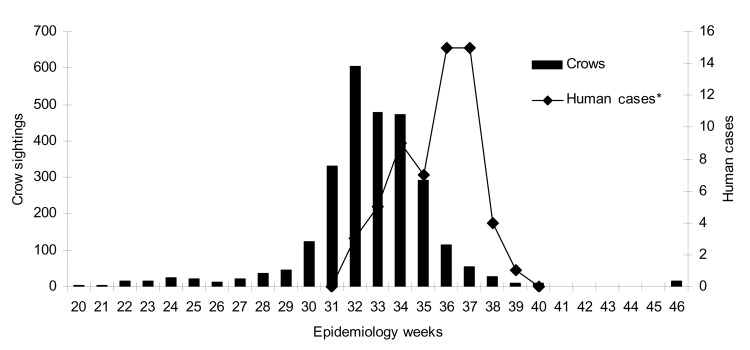
Reported human West Nile virus disease cases and dead crow sightings in Halton Region, May to November 2002, by epidemiology week.

**Figure 2:**
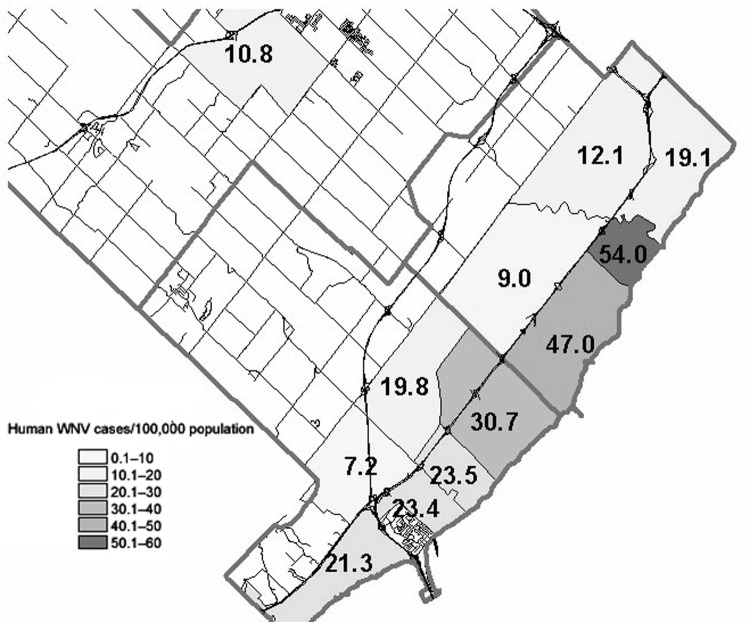
Calculated incidence of human West Nile virus (WNV) cases in south Halton, 2002. The incidence was 47 cases per 100,000 in the L6L forward sortation area (FSA) and 54 cases per 100,000 in the LKL FSA.

## The Study

The survey was conducted from March to April 2003. Households in the L6L and L6K FSAs of south Oakville were selected with random digit dialing. Within households, a randomly selected household member >18 years of age was invited to participate. Given that pediatric neuroinvasive disease is rare, children were excluded (*2*). The 2001 census population of these areas that was >18 years of age was 30,467.

After verbal consent was obtained, respondents were administered a standardized telephone survey. Survey data were collected for respondents who resided in the study area from July 1 to September 30, 2002. Single serum samples were collected from March 23 to June 5, 2003 (specimen collection was interrupted from March 29 to April 16 because of severe acute respiratory syndrome), from persons who had completed the survey. Respondents were unaware of their serologic status at the time of the telephone interview, which reduced the possibility for recall bias. Samples were collected and stored at –70°C until they were tested. Each sample was tested with Centers for Disease Control and Prevention WNV enzyme immunoassay immunoglobulin (Ig) G. Reactive samples were forwarded to Health Canada's National Viral Zoonotic Laboratory in Winnipeg for plaque reduction neutralization tests (PRNT) against West Nile, dengue, and St. Louis encephalitis viruses (*3*). Since our case definition relied on IgG, a positive result may have been caused by infection before the outbreak. However, the prevalence would have been low and would not likely affect our results; surveillance for WNV in Ontario began in 2000, and no positive clinical specimen was seen until the 2002 outbreak (*4*). The ethics review board at McMaster University approved the study.

Based on an assumed population of 30,500, for a prevalence as low as 1%, a sample of 1,500 allows for 95% confidence interval (CI) from 0.5% to 1.5%, and for a prevalence as high as 4% the sample allows 95% CI from 3% to 5%. Initially, 1,500 persons completed the survey, but not all consented to provide a blood sample. As a result, an additional 150 persons were surveyed in April of 2003 to achieve the required sample. Of the 1,650 persons surveyed, 1,505 (91%) consented to provide a blood sample. This fraction represented 25% of persons initially contacted about the study. No significant differences were found in demographic characteristics, so the 2 groups were pooled for subsequent analysis. Because our sample did not correspond in age to the 2001 population (Table 1), we standardized our sample by using age-specific WNV seroprevalences.

**Table 1:** Age and sex of south Oakville, Ontario, survey respondents compared to 2001 census population

Characteristic	Respondents, n (%) (N = 1,650)	No. 2001 population age >18 years, n (%) (N = 30,467)
Sex		
Female	827 (50)	16,015 (53)
Male	823 (50)	14,452 (47)
Age (y)		
18–24	31 (2)	4,045 (13)
25–44	404 (24)	10,740 (34)
45–64	679 (41)	9,465 (30)
>65	531 (32)	7,510 (24)
Education		
Completed high school	1,519 (92)	27,040 (93)
Did not complete high school	116 (7)	2,085 (7)
No answer	15 (1)	

To assess risk factors for WNV infection, we conducted a univariate analysis with chi-square test to assess categorical variables and Student *t* test to assess differences between infected and uninfected persons. Multivariable analysis with logistic regression was performed by using a backwards, stepwise approach, selecting 1 variable from each category to include in the model (indoor exposures, outdoor exposures, personal behavior, source-reduction behavior) if the p value was <0.10.

Forty-six (3.1% [95% CI 2.2%–4.0%]) of the 1,505 persons who provided a blood sample tested positive for WNV IgG, which was confirmed by PRNT. Two (6%) respondents 18–24 years of age, 7 (2%) respondents 25–44 years of age, 26 (4%) respondents 45–64 years of age, and 11 (2%) respondents >65 years of age were infected. In addition to the 46 participants, 14 persons were positive for WNV by IgG enzyme-linked immunosorbent assay but were negative by PRNT. Of these, 11 showed evidence of dengue IgG on PRNT confirmatory testing. No positive respondent had evidence of antibodies to St. Louis encephalitis virus on PRNT testing. The overall estimate of 3.1% did not change based on the 2001 census after adjusting for age.

Within the 2 FSAs from which the sample was drawn were 6 patients with encephalitis (all hospitalized), 5 with meningitis (1 hospitalized), and 8 with WNV fever (1 hospitalized). The calculated rate of WNV illnesses was 47 per 100,000 population in the L6L area and 54 per 100,000 in the L6K area (Figure 2). Cases were defined by the attending physician's diagnosis. No cases of meningitis or encephalitis were seen in persons <50 years of age. Five cases of meningitis and 1 case of encephalitis were seen in persons 50–64 years of age; 2 cases of encephalitis were seen among those 65–74 years, and 3 cases of encephalitis were seen in persons >75 years. Cases were ascertained by the Halton Region Health Department, which did epidemiologic follow-up on all patients with positive WNV serologic results. If we extrapolate the 2.2%–4.0% range to the entire population of adults in the areas studied (30,467), an estimated 670–1,219 persons were infected with WNV in the L6L and L6K areas in the summer of 2002. The ratio of persons with severe illness (defined as meningitis or encephalitis) to asymptomatic or mild cases is, therefore, 1:85 (95% CI 1:60–1:110).

Results of the univariate analysis to assess modifiable risk factors for infection are shown in Table 2. Having an open deck or unscreened porch, time spent outside at dusk or dawn on a work day, time spent outside at dusk or dawn on a nonwork day, and total time spent outside on a nonwork day were associated with WNV infection. Personal behavior associated with WNV infection included rarely or never avoiding areas where mosquitoes are likely to be a problem, rarely or never avoiding going outdoors, and rarely or never wearing long sleeves or long pants when outdoors. However, when >2 personal risk reduction behavior traits were followed, the effect was protective.

**Table 2:** Risk factors for West Nile virus (WNV) infection among household members in south Oakville*

Characteristic	No. (%) respondents or mean (SD)		
	Seropositive (n = 46)	Seronegative (n = 1,459)	OR (95% CI), p value
Indoor exposures			
Open deck or unscreened porch on home	40 (87)	1074 (74)	2.36 (0.99–6.9), 0.04
Tears in screens	12 (26)	343 (24)	1.14 (0.55–2.32), 0.69
Mosquitoes in home >1×/wk	10 (22)	314 (22)	1.01 (0.46–2.14), 0.98
Outdoor exposures			
Time outside at dusk or dawn on work day (h)	2.7 (1.5)	2.1(1.4)	1.32† (1.09–1.58), 0.004
Total time outside on a work day (h)	6.01 (3.9)	5.0 (3.4)	1.08† (1.00–1.16), 0.066
Time spent outside at dusk or dawn on a nonwork day (h)	3.1 (1.9)	2.2 (1.3)	1.48† (1.23–1.78), 0.001
Time total outside on a nonwork day (h)	8.2 (4.5)	6.7 (3.4)	1.13† (1.04 to 1.22), 0.003
Personal behavior			
Rarely or never avoid areas where mosquitoes are likely to be a problem	30 (65)	685 (47)	2.11 (1.10–4.08), 0.015
Rarely or never avoid going outdoors	43 (93)	1,190 (82)	3.2 (1.01–16.20), 0.041
Rarely or never wear long sleeves or long pants when outdoors	30 (65)	715 (49)	1.94 (1.01–3.76), 0.031
Rarely or never wear mosquito repellent when outdoors >30 min	31(67)	944 (65)	1.12 (0.58–2.19), 0.73
Practice >2 personal protective behavior traits‡	19 (41)	894 (61)	0.44 (0.23–0.83), 0.005
Source-reduction behavior			
Drain objects that may collect water	13 (28)	457 (31)	0.86 (0.43–1.71), 0.65
Check and clean gutters	29 (63)	1,009 (69)	0.75 (0.40–1.45), 0.36
Use bug lamps/bug zappers	7 (15)	132 (9)	1.80 (0.67–4.17), 0.156
Practice >2 source-reduction behavior traits	30 (65)	1,044 (72)	0.74 (0.38–1.43), 0.33

*OR, odds ratio; CI, confidence interval. †Odds of WNV infection per hour spent outdoors. ‡Avoiding mosquitoes, wearing long sleeves and long pants, using mosquito repellent.

The following variables were entered in the multivariate model: open deck or unscreened porch, time spent outside at dusk or dawn on a nonwork day, and practicing >2 personal protective behavior traits. Time spent outside at dusk or dawn on a nonwork day (adjusted odds ratio [OR] 1.47 per hour, 95% CI 1.22–1.8, p = 0.001) and practicing >2 personal protective behavior traits (adjusted OR 0.46, 95% CI 0.25–0.84, p = 0.011) were kept in the final model.

## Conclusions

We found in multivariable analysis that respondents who practiced >2 personal protective behavior traits (avoidance of exposure to mosquitoes, wearing long sleeves and pants, using mosquito repellent) had »50% reduction in risk of infection. We also found that time spent outside at dusk or dawn on a nonwork day was a significant risk factor for WNV infection, which is consistent with findings from a previous report (*1*). Finding mosquitoes in the home was not associated with WNV infection, as it was in a previous report (*5*). The seroprevalence in Oakville in 2002 (3%) was within the range of previous reports (*1**,**6**,**7*).

Given the emerging evidence on the long-term sequelae of WNV infection (*8**–**13*), preventing WNV infection is a public health priority. This study is the first to provide evidence to support the benefit of personal protective behavior in reducing risk for WNV infection.
